# Advances in the use of structural and diffusion magnetic resonance imaging for characterizing SCD and MCI due to Alzheimer’s disease

**DOI:** 10.3389/fnins.2025.1596459

**Published:** 2025-08-20

**Authors:** Hao Yang, Cheng Dong, Ying Cai, Mingming Zhao, Junfang Liu, Shizhu Bian, Xiaohan Ding

**Affiliations:** ^1^The 940^th^ Hospital of Joint Logistic Support Force of Chinese People’s Liberation Army, Lanzhou, Gansu, China; ^2^The First School of Clinical Medical, Gansu University of Chinese Medicine, Lanzhou, Gansu, China; ^3^School of Medicine, Northwest Minzu University, Lanzhou, Gansu, China; ^4^The Second Affiliated Hospital of the Army Military Medical University, Chongqing, China

**Keywords:** subjective cognitive decline, mild cognitive impairment, structural magnetic resonance imaging, sMRI, diffusion tensor imaging, DTI

## Abstract

Alzheimer’s disease (AD) has become a great concern for society in general and clinicians specifically because of its high morbidity, relative lack of awareness of its characteristics, and low diagnosis and treatment rates. Worldwide, there is a lack of effective treatments for slowing the progression of AD in clinical practice. Thus, the management of patients in the preclinical phase of AD (PPAD) has been identified to be highly important for addressing this concern. PPAD is considered a preclinical manifestation of the early stages of AD and includes subjective cognitive decline (SCD) and mild cognitive impairment (MCI). Developments in magnetic resonance imaging (MRI) technology have led to its demonstration of great potential in the early identification and progression monitoring of PPAD. Thus, in this review, we summarized the concepts, principles and applications of structural and diffusion MRI in the identification of PPAD to provide potential imaging markers that can be used by clinicians in clinical practice.

## 1 Introduction

Alzheimer’s disease (AD) is an age-related neurodegenerative disorder characterized by cognitive impairment (CI), reduced functional capacity, and non-cognitive neuropsychiatric symptoms. A large clinical epidemiological survey published in 2020 revealed an estimated age-adjusted and sex-adjusted prevalence of AD among individuals of 3.9%; moreover, it is estimated that 9.83 million people aged 60 years or older in China have AD (Jia et al., 2020). AD is characterized by a high morbidity; as the population ages, the number of people with AD is expected to continue to increase, resulting in an increasing burden of the disease. However, current drugs commonly used for managing AD, including cholinesterase inhibitors and N-methyl-*D*-aspartate receptor antagonists, address disease symptoms and are not effective at delaying disease progression (Mangialasche et al., 2010; Nordberg, 2006). AD has an insidious onset and an aggressive course of progression; it is characterized only as mild CI in its early stages, a condition that is frequently difficult for both the patient and their family to notice, delaying the diagnosis and treatment of the disease. Therefore, early identification of the preclinical phase of AD (PPAD) and timely intervention are considered effective means for reducing the disease burden of AD.

A significant percentage of individuals complain that their cognitive abilities have declined from previous levels despite demonstrating normal results on objective neuropsychological tests; this phenomenon is defined as subjective cognitive decline (SCD). The stage following SCD, called mild cognitive impairment (MCI), is characterized by impairment in one or more cognitive domains according to neuropsychological measurements and slight impairment in complex instrumental daily living skills but retention of the ability to live independently; this stage also does not met the diagnostic criteria for AD. Clinical and basic research has suggested that SCD and MCI form a continuum of processes in the onset of AD. Furthermore, SCD and MCI are also considered to constitute the PPAD. Although SCD and MCI are heterogeneous concepts that can be induced by many conditions other than AD (Jessen et al., 2014), including normal ageing, some psychiatric conditions, other neurological and medical disorders and substance abuse, in this review, we specifically focused on SCD and MCI due to AD, excluding all studies that addressed these other etiologies.

A survey of individuals aged 75 years or older revealed that approximately 73.8% manifested SCD (van Harten et al., 2018). Additionally, the overall prevalence of MCI, a type of PPAD and the most common cause of SCD, is approximately 15.5% (Jessen et al., 2014). Previous studies have reported that approximately 12 to 27% of SCD patients had progressed to MCI after 4 years of follow-up in a longitudinal survey, whereas 14% of them had developed dementia. Furthermore, the probability of conversion to dementia was twice as high among patients with SCD as among older adults without SCD (Mitchell et al., 2014; Wolfsgruber et al., 2017). The proportion of patients whose MCI progresses to AD is approximately 10–15%, and the risk of conversion to AD dementia within 5 years is greater than 50% (Gauthier et al., 2006). Therefore, it is important to shift the intervention and management window for AD to the periods corresponding to SCD and MCI in order to maximize both social and economic benefits.

Numerous studies have shown that imaging techniques can detect alterations in brain structure and function *in vivo* during the asymptomatic phase of AD (Habib et al., 2017; Mak et al., 2017). Abnormal deposition of β-amyloid and tau proteins, gray matter atrophy, white matter destruction, and deficits in brain function have been demonstrated to be associated with CI (Lista et al., 2015; Rabin et al., 2017; Sun et al., 2015). Imaging can provide non-invasive and objective evaluation metrics for the assessment of CI. With the rapid development of imaging techniques, their great potential for application in the preclinical identification of AD will become a topic of great interest in clinical practice.

Thus, in this review, we summarize research advances in the application of structural magnetic resonance imaging (sMRI) and diffusion MRI (dMRI) in the identification of SCD and MCI to explore the trajectory of progression between these two stages and provide morphological and diffusion evidence for the identification of PPAD.

## 2 The basic concepts of sMRI

sMRI is a medical imaging modality that combines the principles of nuclear MRI and radiofrequency wave technology to detect structural changes in tissues. sMRI can not only display the morphology of brain tissues but also reflect changes in the volumes, thicknesses and surface areas of brain nuclei. In addition, patients scanned with sMRI avoid exposure to radiation, as this technique involves multidirectional and multiparameter imaging. Compared with positron emission tomography (PET), sMRI demonstrates advantages including radiotracer-free imaging. Similarly, compared with resting-state functional MRI (fMRI), sMRI is not restricted by large amounts of noise interference. Owing to these advantages, sMRI analysis techniques have been widely used in studies on PPAD. These techniques can be broadly grouped into voxel-based morphometry (VBM) and surface-based morphometry (SBM).

Briefly, VBM is a highly comprehensive, objective and accurate method for evaluating the morphological changes in brain tissue in the PPAD, and is typically used to measure nuclear volume. VBM is also employed as a whole-brain technique for characterizing the differences between the regional volumes and tissue concentrations of two groups from structural MRI scans (Ashburner and Friston, 2000). Prior to the advent of VBM, manual delineation of the region of interest was the gold standard for measuring the volumes of brain structures. However, compared with the region-of-interest approach, VBM presents many advantages and has been more widely used in the neuroimaging community. Moreover, VBM has comparable accuracy to manual volumetry (Wang W. Y. et al., 2015), with several studies showing good correspondence between the two techniques (Davies et al., 2009; Giuliani et al., 2005), providing confidence in the biological validity of the VBM approach.

Unlike VBM, SBM tends to be used to measure the surface area and thickness of nuclei. It quantifies the changes in gray matter structure under various pathological conditions by calculating the morphological parameters of the brain tissue, such as thickness, surface area and gray matter volume (Goto et al., 2022). SBM can not only quantify the atrophy rate of brain tissue in AD patients but also reflect their pathological and biochemical changes. The cortical thickness and volume as determined via SBM are correlated with the extent of neurofibrillary tangles (Schwarz et al., 2016), and the degree of brain shrinkage can directly reflect neuronal degeneration and is related to the amount of filament light chain protein in cerebrospinal fluid (Allison et al., 2019). In this way, SMB can provide more information than VBM regarding cognitive changes.

### 2.1 Application of sMRI in SCD

Asymmetry between the right and left hippocampus and amygdala is considered a biomarker of SCD in numerous voxel analysis studies (Yue et al., 2018). In addition to asymmetry between cerebral areas, significant reductions in hippocampal volume in individuals with SCD at baseline have been reported in other studies (Hafkemeijer et al., 2013; Kim et al., 2013; Rogne et al., 2016; Sánchez-Benavides et al., 2018). Identical results have been reported in longitudinal follow-up studies, indicating that the hippocampal volume decreases at a rate of 1.9% per year in people with SCD (Cherbuin et al., 2015; Nunes et al., 2010). Hippocampal atrophy manifests mainly as a significant reduction in the volume of gray matter in the tail of the bilateral hippocampus and enlargement of the bilateral paracentral lobules (Liang et al., 2020). However, other studies have not reported significant hippocampal atrophy (Platero et al., 2019). For example, a study revealed significant cortical atrophy in their bilateral parahippocampus and perirhinal and the left entorhinal cortices (Fan et al., 2018). The other study demonstrated that the volume of the entorhinal cortex in individuals with SCD was smaller than normal cognitive participants (Ryu et al., 2017). These differences across studies may be related to the heterogeneity among individuals included in those studies, which in turn may be related to the sources from which these subjects were recruited (Pini and Wennberg, 2021) and the enrolment criteria (Perrotin et al., 2017) applied in the studies. However, the hippocampal volume is insufficient and lacks specificity as an independent diagnostic basis for SCD. For example, patients with depression may also present with reduced hippocampal volume and CI (Frodl et al., 2002; Sheline, 2003). Furthermore, the hippocampus is highly susceptible to ageing and ageing-related changes (Bettio et al., 2017). In addition, several studies have investigated differences in volume in numerous subcortical areas, including the cholinergic basal forebrain nuclei and hippocampal subregions, between individuals with SCD and normal cognitive controls (NCs). These findings consistently suggest that SCD is associated with a significant reduction in the cholinergic basal forebrain and hippocampal CA1 volumes with respect to NCs (Cantero et al., 2016; Perrotin et al., 2015; Scheef et al., 2019; Zhao et al., 2019).

Other methods for identifying and evaluating SCD have focused mainly on surface-based analyses. In addition to reduced subcortical volume, surface-based analyses have demonstrated that a thinner cortex, particularly in the temporoparietal lobe, is associated with faster memory deterioration and an increased risk of disease progression in patients with SCD than in NCs (Meiberth et al., 2015; Schultz et al., 2015; Verfaillie et al., 2016; Verfaillie et al., 2018). Another study revealed that both SCD and MCI patients presented with a reduced volume in the left inferior parietal lobe (IPL), while SCD patients also presented with morphological changes in the right inferior temporal gyrus (ITG), right insula, and right amygdala (Song et al., 2024). Another study showed that compared with NCs, patients with MCI presented with predominantly left-sided surface morphology changes across various brain regions, including the transverse temporal gyrus, superior temporal gyrus, insula, and pars opercularis. Patients with SCD, meanwhile, exhibited relatively slight surface morphological changes, mainly in the insula and deltoid (Yang J. et al., 2023). A prospective study revealed that larger volumes and lower amyloid loads in the right and left parietal lobes at baseline were associated with a greater probability of cognitive recovery in SCD patients, providing clinicians with key factors associated with cognitive improvement (Na et al., 2024). In another study, the overall amyloid loads of the right hemisphere and specifically the right temporal lobe cortex of amyloid-positive SCD patients were negatively correlated with the gray matter volume, providing a pathological perspective for validating SCD as a preclinical stage of AD (Wang et al., 2022). We summarized the main studies of structural MRI studies in SCD in Table 1.

**TABLE 1:** Summary of structural MRI studies in SCD.

Authors	Modality	Design	Sample	Main findings
Yue et al., 2018	T1 MRI	Cross-sectional	SCD = 111 MCI = 30 NC = 67	SCD showed significance decreased right hippocampal and amygdala volume than control. Asymmetry pattern: hippocampus: left-larger-than-right; amygdala: left-less-than-right
Hafkemeijer et al., 2013	T1 MRI	Cross-sectional	SCD = 25 NC = 29	Gray matter volume reductions in these area: hippocampus, anterior cingulate cortex (ACC), medial prefrontal cortex, cuneus, precuneus, and precentral gyrus
Kim et al., 2013	T1 MRI	Cross-sectional	SCD = 90 NC = 28	The volumes of hippocampus and amygdala in SCD were significantly smaller than NC.
Rogne et al., 2016	T1 MRI	Cross-sectional	SCD = 25 MCI = 115 NC = 58	SCD and MCI had significantly larger lateral ventricles and smaller hippocampal volumes than controls.
Sánchez-Benavides et al., 2018	T1 MRI	Cross-sectional	SCD = 572 NC = 2098; SCD+ = 253 SCD− = 319	The SCD + group showed lower left total intracranial volume- adjusted hippocampal volumes than the non-SCD (*p* = 0.004) and SCD– (*p* = 0.017) groups in unadjusted comparisons.
Cherbuin et al., 2015	T1 MRI	longitudinal study	W1 SCD = 70 W2 SCD = 56 W1 + W2 SCD = 39 NC = 218	SMD at baseline was not a significant predictor of hippocampal atrophy. However, SMD at follow-up was associated with greater hippocampal atrophy.
Nunes et al., 2010	T1 MRI	longitudinal study	SCD = 15 MCI = 17 NC = 11	SCD suffered a decline in the total hippocampal volume.
Liang et al., 2020	T1 MRI	Cross-sectional	SCD = 35 NC = 32	Individuals with SCD showed significant gray matter volume decreases in the bilateral hippocampal tails and enlargement of the bilateral paracentral lobules.
Platero et al., 2019	T1 MRI	longitudinal study	SCD = 87 MCI = 137 AD = 13 NC = 70	We did not find significant differences in normalized hippocampal volumes between the NC and SCD groups.
Fan et al., 2018	T1 MRI	Cross-sectional	SCD = 43 aMCI = 44 NC = 34	SCD subjects showed significant cortical atrophy in their bilateral parahippocampus and perirhinal and the left entorhinal cortices but not in their hippocampal regions.
Ryu et al., 2017	T1 MRI DTI	Cross-sectional	SCD = 18 NC = 27	Individuals with SMI had lower entorhinal cortical volumes than control participants, but no differences in hippocampal volume.
Perrotin et al., 2015	T1 MRI	Cross-sectional	SCD = 17 AD = 21 NC = 40	Both patient groups showed significant TIV-normalized volume decrease in hippocampus global volume and in CA1 and subiculum subfields as well as in the other subfield in AD compared to controls.
Cantero et al., 2016	T1 MRI	Cross-sectional	SCD = 47 NC = 48	Individuals with SMC exhibited significantly lower volumes of CA1, CA4, dentate gyrus, and molecular layer.
Scheef et al., 2019	T1 MRI	Cross-sectional	SCD = 24 NC = 49	The SCD cases showed a significant total volume reduction of the cholinergic basal forebrain nuclei.
Zhao et al., 2019	T1 MRI	Cross-sectional	SCD = 35 aMCI = 43 AD = 41 NC = 42	CA1, subiculum, presubiculum, molecular layer and fimbria showed the trend toward significant volume reduction among four groups with the progression of Alzheimer’s disease.
Meiberth et al., 2015	T1 MRI	Cross-sectional	SCD = 41 NC = 69	Cortical thickness reduction was observed in the SCD group compared to controls in the left entorhinal cortex.
Schultz et al., 2015	T1 MRI	Cross-sectional	SCD = 77 NC = 184	Individuals with SCDs had significant cortical thinning in the entorhinal, fusiform, posterior cingulate, and inferior parietal cortices.
Verfaillie et al., 2018	T1 MRI	longitudinal study	SCD = 233	A faster subsequent rate of memory loss was associated with thinner cortex of the frontal, temporal, and occipital cortices.
Song et al., 2024	T1 MRI DWI	Cross-sectional	SCD = 37 MCI = 28 NC = 42	Both SCD and MCI showed decreased volume in left inferior parietal lobe (IPL), while SCD showed altered morphologies in the right inferior temporal gyrus (ITG), right insula and right amygdala.
Yang J. et al., 2023	T1 MRI	Cross-sectional	SCD = 62 MCI = 97 NC = 70	Compared to NC, patients with MCI exhibited predominantly left-sided surface morphological changes in various brain regions, including the transverse temporal gyrus, superior temporal gyrus, insula, and pars opercularis. SCD patients showed relatively minor surface morphological changes, primarily in the insula and pars triangularis.
Na et al., 2024	T1 MRI PET	longitudinal study	SCD = 120	This study showed a lower frequency of amyloid PET positivity and larger volumes in the left and right superior parietal lobes in subjects with improved memory function.
Wang et al., 2022	T1 MRI PET	Cross-sectional	SCD = 93	This study found a negative relationship between global amyloid load and GM volume in the right hemisphere (*r* = 0.441, *p* = 0.012) and right temporal cortex (*r* = 0.506, *p* = 0.003) in the amyloid-positive group.
Fu et al., 2021	T1 MRI	Cross-sectional	SCD = 35 aMCI = 43 AD = 41 NC = 42	Structural covariance networks (SCNs) seeding from the default mode network (DMN), salience network, subfields of the hippocampus, and cholinergic basal forebrain showed increased structural covariance at the early stage of AD (referring to aMCI) and decreased structural covariance at the dementia stage (referring to AD).
Xu et al., 2022)	T1 MRI rs-MRI	Cross-sectional	SCD = 53 NC = 65	Gray matter volume (GMV) results demonstrated decreased GMV in the bilateral ventrolateral prefrontal cortex (vlPFC) and right insula in patients with SCD relative to NCs. Differences were observed in the structural covariance of several regions mainly comprising the left anterior cingulate cortex, bilateral precuneus, left syrinx, and left midoccipital cortex.

Notably, a growing number of studies have measured gray matter volumes among brain regions by employing a structural covariance network approach (de Schipper et al., 2017; Drenthen et al., 2022; Watanabe et al., 2020), in which the gray matter covariance is examined by mapping gray matter correlations across the brain to seed regions. The structural covariance approach is essentially a correlation analysis of cross-sectional morphometric imaging data in which gray matter shrinkage is measured in common processes between brain regions (Alexander-Bloch et al., 2013). Gray matter covariances may provide more neural information than the volumes of individual brain regions. For example, structural covariances have been reported to reflect the effects of brain development and structural plasticity (Duan and Wen, 2023). Studies on structural covariance patterns have revealed reduced structural covariance (Tsai et al., 2023) and weakened connectivity strength in SCD patients (Fu et al., 2021). Furthermore, reduced gray matter volumes in the bilateral ventral lateral prefrontal cortex and right insula in SCD patients relative to NCs have also been identified. Take the above-mentioned area as the ROIs, there was an abnormal structural association between the left ventrolateral prefrontal cortex (vlPFC) and regions, including the left anterior cingulate cortex (ACC), right vlPFC, left insula, and right dorsolateral prefrontal cortex (dlPFC) in the patients with SCD compared to NCs. The left ACC, left middle occipital cortex (MOC), bilateral fusiform and bilateral precuneus (PCUN) showed decreased structural covariance with the right vlPFC in patients with SCD compared to NCs. For the right insula ROI, decreased structural covariance was observed between the right insula and regions including the right median cingulate cortex (MCC), right PCUN, right MOC, left hippocampus, left thalamus, and right ACC (Xu et al., 2022). Since the pathology of AD (such as Aβ and Tau deposition) may affect the distal brain regions through neural connections, leading to the disruption of the covariance network, the decreased covariance association may predict the early damage of the memory-related network. In addition to traditional methods of analysis, an increasing number of studies are employing novel analytical modalities to process preclinical AD imaging findings, opening new avenues for exploring pathophysiological alterations in AD.

### 2.2 Application of sMRI in MCI

VBM-based studies have revealed that an abnormal reduction in hippocampal volume can be observed 8 years prior to a clinical diagnosis of AD (Jia et al., 2024). Furthermore, the hippocampus continues to atrophy as the disease progresses, making its volume one of the most well-established imaging biomarkers of the AD disease spectrum (Bayram et al., 2018). Similarly, atrophy of the hippocampus has been observed in patients with MCI, in addition to substantial atrophy of the internal olfactory cortex (Pennanen et al., 2004; Teipel et al., 2006), which may be related to the pathological mechanisms underlying cognitive dysfunction. In addition, atrophy of the nucleus accumbens and the right caudate nucleus has been reported to mark the transition from MCI to AD, whereas volume reductions in the right thalamus and bilateral caudate nucleus have been associated with CI in AD patients (Tuokkola et al., 2019). In conclusion, we can use neuroimaging techniques to assess the volume changes of voxels that are significantly associated with MCI. Among them, structural MRI has demonstrated the greatest benefits, as it can achieve excellent soft tissue contrast and high spatial resolution, which are important for detecting AD. Although structural changes revealed on sMRI cannot be used as a specific feature of MCI, they provide a biased basis for the evaluation of this condition. The combination of sMRI and automated diagnostic methods provides a new perspective for predicting the transformation of MCI to AD (Garg et al., 2023).

SBM-based analyses, meanwhile, have suggested that cortical thickness is associated with AD severity and CI (van Oostveen and de Lange, 2021). Gray matter atrophy, mainly in the medial temporal lobe and subsequently extending along the temporo-parietal-frontal lobes to the remaining cortex, has been reported in patients with early AD (Pini et al., 2016). Researchers have suggested that temporal occipital cortex atrophy could be a sensitive and specific marker of pathological changes in AD (Mao et al., 2022). Medical researchers have compared the temporal lobe volumes of NCs and MCI and AD groups and reported that compared with those of the NCs, the hippocampal volumes of the MCI and AD groups were significantly reduced, whereas temporal lobe neocortical atrophy was identified only in the AD group (Convit et al., 1997). Follow-up studies have revealed that MCI patients who progress to AD are more likely to gradually develop temporal neocortical atrophy than patients with stable MCI, suggesting temporal neocortical atrophy may be a biomarker of disease progression (Convit et al., 2000). Fellow Li reported that reductions in cortical thickness in individuals with MCI starts in the temporal lobe, but the range of thickness changes gradually expands as the disease progresses. In addition, reductions in cortical thickness have been positively associated with reduced scores on the Mini-Mental State Exam (MMSE). Thus, changes in temporal cortex thickness may reflect alterations in cognitive functioning in patients with AD (Li et al., 2022). A longitudinal study revealed that the bilateral temporal cortex was significantly thinner in AD patients than in NCs, whereas this difference was less pronounced between MCI patients and NCs (Bachmann et al., 2023). In addition, Yuanlin revealed that the gray matter volumes in the left superior and right middle temporal gyri were lower in AD patients than in MCI patients, suggesting that gray matter atrophy in the lateral temporal lobe may help differentiate MCI and AD (Yuanlin et al., 2021). A large body of evidence suggests that atrophy of the medial temporal lobe and changes in the cortical thickness of the lateral temporal lobe are early biomarkers of AD-associated CI (Table 2).

**TABLE 2:** Summary of structural MRI studies in MCI.

Authors	Modality	Design	Sample	Main findings
Jia et al., 2024	T1 MRI	longitudinal study	NC = 648 AD = 648	Hippocampal volume in the Alzheimer’s disease group diverged from it in the cognitively normal group 8 years before diagnosis.
Pennanen et al., 2004	T1 MRI	Cross-sectional	MCI = 65 AD = 48 NC = 59	The hippocampus and ERC were significantly reduced in MCI and AD.
Teipel et al., 2006	T1 MRI	Cross-sectional	probable AD = 34 NC = 22	Volumes of medial temporal lobe structures were significantly smaller in probable AD patients than in controls with exception of the left entorhinal cortex.
Tuokkola et al., 2019	TBM-MRI	Cross-sectional	MCI = 38 AD = 58 NC = 58	Atrophy of the DGM structures, especially the thalamus and caudate nucleus, is related to cognitive impairment in AD.
Mao et al., 2022	T1 MRI	Cross-sectional	probable AD = 111	The volume of the temporal-parietal-occipital cortex was most strongly associated with cognitive decline in group analysis.
Convit et al., 1997	T1 MRI	Cross-sectional	MCI = 22 AD = 27 NC = 27	By the time impairments are sufficient to allow a diagnosis of DAT to be made, in addition to the medial temporal lobe volume reductions, the lateral temporal lobe is also showing volume reductions, most saliently involving the fusiform gyrus.
Convit et al., 2000	T1 MRI	longitudinal study	MCI = 20 NC = 26	The medial occipitotemporal and the combined middle and inferior temporal gyri may be the first temporal lobe neocortical sites affected in AD; atrophy in these areas may herald the presence of future AD among non-demented individuals.
Li et al., 2022	T1 MRI	Cross-sectional	aMCI = 48 AD = 33 NC = 33	In the AD-aMCI groups, the brain regions with reduced cortical thickness primarily included the bilateral superior temporal gyrus, bilateral transverse temporal gyrus, bilateral insula, bilateral posterior cingulate gyrus, right temporal pole, right entorhinal cortex, right fusiform gyrus, and right paracentral gyrus. The cortical thickness was positively correlated with the MMSE and MoCA scores.
Bachmann et al., 2023	T1 MRI	longitudinal study	MCI = 211 AD = 96 NC = 165	Bilateral temporal cortex thickness was significantly thinner in the AD patients compared to the NC group, whereas bilateral temporal cortex thickness was less thinned in the aMCI patients compared to the NC group
Rechberger et al., 2022	T1 MRI	longitudinal study	AD = 25 NC = 25	The surface area of temporal lobe, superior frontal gyrus, and anterior cingulate cortex in the AD group was significantly lower than that of the NC group.
Serra et al., 2022	T1 MRI	Cross-sectional	aMCI = 104 AD = 110 NC = 64	The degree of cortical folding is related to cognitive function in older adults
Núñez et al., 2020	T1 MRI	Cross-sectional	MCI = 384 AD = 178 NC = 223	An increased degree of folding of the insular cortex was specifically associated with better memory function and semantic fluency, only in AD patients.

sMRI analysis based on the calculation of surface area is also frequently employed as a strategy in AD-related CI studies. According to Rechberger and colleagues, the surface areas of the temporal lobe, superior frontal gyrus, and anterior cingulate cortex in the AD group are significantly lower than those in the NC group, which suggests that a decrease in the surface area of the cerebral cortex may be related to the reduction in cognitive function observed in AD patients (Rechberger et al., 2022). The cerebral cortex is well characterized by substantial gyrification; consequently, the cortical surface is mostly hidden in the sulci and lateral fossa. Recent studies have shown that the fractal dimension (FD), gyration index (GI), and sulcus depth (SD) can be used as valuable measurements of the complexity of cortical folding (Qin et al., 2022). Other studies have indicated that the degree of cortical folding is related to the cognitive functioning of older adults (Serra et al., 2022). A longitudinal study revealed that reductions in the FD, GI, and SD were associated with cognitive decline in AD patients. Furthermore, overall changes in the FD, GI, and SD were more pronounced in patients with AD than in those with amnestic MCI (aMCI), whereas changes in the FD were associated with cognitive ability only in AD patients but not in aMCI patients (Bachmann et al., 2023). As shown in another study, a greater insular cortical GI is strongly associated with better memory function and semantic fluency in AD patients, whereas this association was much less pronounced in MCI patients and was not present in NCs. This could suggest that as the disease progresses, the increasingly atrophic medial temporal lobe is unable to maintain memory function, requiring the insular cortex to gradually take it over (Núñez et al., 2020).

In conclusion, morphology-based analyses suggest that hippocampal atrophy, as well as medial temporal lobe atrophy, is a common pathological change in PPAD and that the degree of atrophy increases as SCD progresses to MCI. Moreover, as the patient’s cognitive deficits worsen, cortical atrophy advances from the temporal lobe to the parietal lobe, then frontal lobe, and finally spreads to the entire cerebral cortex. In addition, asymmetry of the hippocampus and amygdala, cholinergic basal ganglia, nucleus accumbens, caudate nucleus, and thalamus are potential biomarkers of preclinical AD. Despite its relative novelty, structural covariance analysis has been gradually applied to preclinical imaging analysis of AD (Alexander-Bloch et al., 2013; Montembeault et al., 2016; Seeley et al., 2009). Compared with other analytical methods, it can better analyze the correlations between the morphologies of different regions of the brain, providing richer evidence of early pathological changes in AD. However, there are still some obstacles to its application. For instance, there are gaps in the comparability between different sample datasets and the reliability of the results for the same subject at different periods and image resolutions. Additional studies are needed to explore and verify the reproducibility of methods based on structured correlation analysis (Table 2).

## 3 The basic knowledge dMRI

Diffusion MRI is an imaging technique that leverages the magnetic resonance signal changes caused by the diffusion anisotropy of water molecules in tissues to visualize the microstructure of these tissues (Silva-Rudberg and Mecca, 2024). Diffusion based techniques have proven to be particularly useful in investigating cerebral infarction, cerebral infections, epidermoid and other cysts, cerebral tumors, and white matter (WM) disorders (Newcombe et al., 2013). Therefore, it is widely utilized in the study of microstructural changes associated with (AD). Diffusion imaging encompasses diffusion tensor imaging (DTI) and more specific imaging techniques derived from DTI. Among them, DTI is the most widely used diffusion imaging technique. The following are the applications of several diffusion imaging techniques in SCD and MCI.

### 3.1 The introduction of DTI

Diffusion tensor imaging (DTI) is commonly used to study the microstructural changes in WM in patients with neurodegenerative diseases, including axonal loss, damage, or demyelination (Alexander et al., 2007). DTI images are usually characterized by parameters including fractional anisotropy (FA), mean diffusivity (MD), axial diffusivity (AD), and radial diffusivity (RD). FA is the most commonly used metric in evaluating DTI data and reflects the directionality and integrity of WM fibers; a reduction in the FA value indicates that the integrity of the WM has been compromised (Chung et al., 2011). The AD represents the diffusion rate of water molecules in the direction of the main axis and can reflect the growth of axons; this value decreases when axons are damaged. The RD represents the diffusion rate of water molecules in the direction perpendicular to the main axis of diffusion and can reflect the formation of myelin sheaths; the value increases as the amount of myelin in the sheaths decreases. Finally, the MD represents the overall diffusion of molecules regardless of direction; an increase in this value suggests an increase in the content of free water molecules in the tissue. DTI analytical techniques can be divided into traditional voxel-based analysis (VBA) and tract-based spatial statistics (TBSS).

Both VBA and TBSS allow the spatial localization of WM fiber bundles for subsequent quantitative analysis. In VBA, the individual diffusion information is first registered to a standard spatial template, which is then segmented on the basis of spatial location via a WM atlas to obtain quantitative information on the corresponding fiber bundles. The VBA method is intuitive, convenient and is not greatly influenced by human subjective, but the results are highly dependent on the accuracy of the registration; if it is incorrect, false positives may occur, and individual differences may be underestimated (Shu et al., 2009). To overcome the shortcomings related to registration in VBA and smooth kernels without uniform values (Jones et al., 2005), several scholars (Smith et al., 2006) have proposed the TBSS analysis method. First, a skeleton is formed in standard space on the basis of the FA chart of each subject, and then the individual dispersion index is projected onto the skeleton for point-by-point statistical analysis. In addition, in TBSS, a WM segmentation template can be applied to obtain the dispersion index of each fiber bundle. TBSS can be performed automatically by some software, resulting in a rapid and convenient analysis. However, this method is not sensitive to the direction of the fibers. Owing to individual differences in the positions of WM fibers and the existence of cross-fibers, mutual interference from fiber bundles is not uncommon in the results.

#### 3.1.1 Application of DTI in SCD

The brains of individuals with SCD have shown significant reductions in FA and increases in the MD, mainly in the hippocampus, internal olfactory cortex, parahippocampal gyrus, leptomeningeal tract, longitudinal fasciculus, and corpus callosum (Brueggen et al., 2019; Li et al., 2016; Ohlhauser et al., 2019; Wang et al., 2012; Yasuno et al., 2015). Another study revealed that the WM connectivity of the left IPL, lateral occipital cortex (LOC) and insular fiber tracts differed between SCD patients and MCI patients. The volume of the left IPL, right LOC, and right amygdala and the diffusivity value of the right LOC fiber tracts were significantly associated with the cognitive functioning of the subjects (Song et al., 2024). Specifically, the Montreal cognitive scores were negatively correlated with the MD in both the inferior cerebellar pedicle and right corticospinal tract. However, degeneration of specific WM tracts is common in the physical cognitive decompensation of SCD (Wei et al., 2021). Scientists have found increased RD and MD values in a wide range of WM tracts among patients with SCD via whole-brain voxel analyses but no significant alterations in FA. This result suggests that both FA and the MD may be important in revealing the early pathological processes involved in AD, with the MD potentially being more sensitive than FA. Genetic risk may exacerbate degeneration in patients with SCD; ApoEε4 carriers in the SCD population have demonstrated lower FA in the splenium and anterior part of the corona radiata than non-carriers did (Lee et al., 2016). Another study categorized SCD patients into high- and low-risk groups on the basis of age, ApoE genotype, Korean MMSE (K-MMSE) recall score, and Seoul Language Learning Test score. Compared with the low-risk group, the high-risk group presented with more severe microstructural disruption and reduced FA in nerve bundles connecting the hippocampus, parahippocampal gyrus, supramarginal gyrus, and part of the temporal lobe (Hong et al., 2016). The studies of DTI in SCD have also been summarized in Table 3.

**TABLE 3:** Summary of DTI studies in SCD.

Authors	Modality	Design	Sample	Main findings
Brueggen et al., 2019	DTI	Cross-sectional	SCD = 98 MCI = 45 AD = 35 NC = 2–76 participants per center;	FA were lower in SCD compared to NC in several anterior and posterior WM regions, including the anterior corona radiata, superior and inferior longitudinal fasciculus, cingulum and splenium of the corpus callosum; MD was higher in the superior and inferior longitudinal fasciculus, cingulum and superior corona radiata.
Li et al., 2016	DTI	Cross-sectional	SCD = 27 MCI = 35 AD = 25 NC = 37	As compared to NC subjects, SCD patients displayed widespread WM alterations represented by decreased FA, increased mean diffusivity, and increased radial diffusivity. In addition, localized WM alterations showed increased axial diffusivity. In the shared WM impairment tracts, SCD patients had FA values between the NC group and the other two patient groups.
Ohlhauser et al., 2019	DTI	Cross-sectional	SCD = 30 NC = 44	The between-group FA analyses revealed diffuse reductions in the SCD group relative to the healthy control group in regions including bilateral corticospinal tracts, superior and inferior longitudinal fasciculi, fronto-occipital fasciculi, corpus callosum, forceps major and minor, hippocampi, anterior thalamic radiations, and the cerebellum. The between-group MD analyses showed diffusely higher MD in the SCD group compared with the control group in regions including bilateral corticospinal tracts, superior and inferior longitudinal fasciculi, superior corona radiata, and corpus callosum.
Wang et al., 2012	DTI	Cross-sectional	SCD = 29 MCI = 28 NC = 35	The mild cognitive impairment group showed lower fractional anisotropy and higher radial diffusivity than controls in bilateral parahippocampal white matter.
Yasuno et al., 2015	DTI	Cross-sectional	SCD = 23 NC = 30	Results revealed significantly lower FA of the superior longitudinal fasciculus at the left external capsule and higher FA in the left cingulum near the hippocampus in SCD subjects compared with non-SCD subjects.
Wei et al., 2021	DTI	Cross-sectional	SCD = 46 NC = 49	Montreal cognitive scores were correlated with MD in the bilateral inferior cerebellar peduncles and right corticospinal tracts.
Lee et al., 2016	DTI	Cross-sectional	SCD = 26 ApoE ε4 carriers = 13 ApoE ε4 non-carriers = 13	ApoE ε4 carriers compared with non-carriers in SMI without WMH showed the atrophy of GM in inferior temporal gyrus, inferior parietal lobule, anterior cingulum, middle frontal gyrus, and precentral gyrus and significantly lower fractional anisotropy WM values in the splenium of corpus callosum and anterior corona radiate.
Hong et al., 2016	DTI	Cross-sectional	SCD = 46 high risk of progression = 19 low risk of progression = 27	The high-risk group had more microstructural disruption shown by lower fractional anisotropy in the hippocampus, parahippocampal gyrus, supramarginal gyrus, and parts of frontotemporal lobes.

DTI, one of the most important forms of MRI, can describe the microstructure of WM through its corresponding tensor model. Consequently, DTI is widely used to understand relevant central nervous system mechanisms and identify appropriate potential biomarkers for the early stages of AD. Moreover, the diffusion features and structural connectomics of specific regions can provide information for aiding in the early recognition of AD. A recent study achieved an accuracy of up to 92.68% in distinguishing SCD patients from normal controls with DTI (Chen et al., 2023).

#### 3.1.2 Application of DTI in MCI

Researchers have also applied the DTI technique to analyze WM microstructural changes in patients with aMCI with different developmental trajectories; the results revealed that the right cingulate gyrus FA values and right hippocampus MD values were particularly sensitive to disease progression, suggesting that these parameters could serve as imaging biomarkers for predicting the developmental trajectories of aMCI patients in the future. Tinney and colleagues also used TBSS to calculate the MD values of the splenium and body of the corpus callosum, the superior corona radiata and the retrolenticular part of the internal capsule in patients with aMCI. They also reported that the MD values were strongly correlated with reductions in cognitive function metrics (Tinney et al., 2023). A previous study revealed fine anatomical changes in the WM of patients with aMCI and AD via VBA, TBSS and fiber bundle auto quantification analysis. The authors reported a regional decrease in FA and an increase in the MD in patients with aMCI, whereas these changes were more widespread throughout the brain in AD patients (Zhang et al., 2019). According to Farrar, MCI patients with high executive ability have more complete WM fiber tracts than those with low executive ability, suggesting that the reduced integrity of WM fiber tracts is associated with reduced executive ability in these patients (Farrar et al., 2018). A study concluded that left hippocampal and cingulate fasciculus FA could differentiate stable from progressive MCI (Marcos Dolado et al., 2019), suggesting that DTI may assist in the early identification of individuals at risk for MCI. The results from a longitudinal study revealed that individuals at risk for MCI were characterized by reduced FA and an increased MD in the WM of the fornix and left parahippocampal gyrus. Furthermore, the WM microstructural changes of MCI patients were associated with disease progression (Zhimei et al., 2017). In patients with MCI, WM microstructural changes in the hippocampus, fornix, and cingulate fasciculus should be assessed during regular follow-up to monitor disease progression in a timely manner. The studies of DTI in SCD have also been summarized in Table 4.

**TABLE 4:** Summary of DTI studies in MCI.

Authors	Modality	Design	Sample	Main findings
Tinney et al., 2023	DTI	Cross-sectional	MCI = 20 NC = 60	It found that the MD increases of the corpus callosum stem, corpus callosum pressure, superior radiocarpal and posterior limb of the internal capsule in patients with aMCI, and it was significantly correlated with cognitive function reduction.
Zhang et al., 2019	DTI	Cross-sectional	MCI = 29 NC = 34	In comparison with NC, AD patients showed widespread FA reduction in 25% (5/20) and MD increase in 65% (13/20) of the examined fiber tracts. The MCI patients showed a regional FA reduction in 5% (1/20) of the examined fiber tracts (right cingulum cingulate) and MD increase in 5% (1/20) of the examined fiber tracts (left arcuate fasciculus).
Farrar et al., 2018	DTI	Cross-sectional	MCI-highEF = 15 MCI-lowEF = 16	The network measures of the high executive ability group demonstrated greater white matter integrity.
Marcos Dolado et al., 2019	DTI	longitudinal study	MCI = 135 NC = 72	The factors MD left hippocampus, FA left cingulate, and FA left hippocampus emerged as predictors of progression.
Zhimei et al., 2017	DTI	longitudinal study	NC = 102	It showed that individuals at risk for MCI had reduced FA and increased MD in the white matter of the fornix and left parahippocampal gyrus, and their white matter microstructural changes were associated with disease progression

The above studies indicate that both SCD and MCI patients present with a reduction in FA and an increase in the MD in the WM tracts of the hippocampus, suggesting that this is a key brain region for recognizing CI. Furthermore, these alterations occurred prior to the time when objective evidence of CI could yet be obtained, and the damage worsened as the disease progressed. The results of DTI-based studies indicate that there is considerable heterogeneity in the WM damage that occurs in both SCD and MCI patients. Thus, more studies with larger sample sizes are needed to better assess this damage.

### 3.2 Application of DTI-derived techniques in PPAD

Although DTI is currently the most widely used technique worldwide for studying the microscopic structural characteristics of WM in the central nervous system (Palacios et al., 2020), it has limitations such as the inability to effectively display crossed or branched fibers, and is susceptible to interference from CSF and free water (Andica et al., 2020). Therefore, a series of DTI derivative techniques have emerged based on the differences in diffusion direction and speed of water molecules in different structures, including Diffusion Kurtosis Imaging (DKI), Free Water DTI (FW-DTI), and Neurite Direction Dispersion and Density Imaging (NODDI).

#### 3.2.1 Application of DKI

Diffusion kurtosis imaging (DKI) more accurately depicts the diffusion of water molecules in complex microenvironments by quantifying the degree of non-Gaussian water diffusion in tissues, thereby detecting the microstructure of biological tissues. DKI extends traditional DTI techniques, providing not only DTI parameters but also kurtosis parameters such as mean kurtosis (MK), axial kurtosis (AK), and radial kurtosis (RK). The kurtosis parameters of DKI are particularly suitable for assessing the microstructural integrity of WM regions with complex fiber arrangements (Raj et al., 2022).

Study involving NC, SCD, and CI groups showed significant differences in mean kurtosis tensor and kurtosis tensor anisotropy among the three groups (Bergamino et al., 2024), suggesting that DKI holds promise for staging AD. A recent study focusing on the hippocampus found that left hippocampal MK effectively distinguishes between SCD and NC, with more pronounced changes in hippocampal MD and MK values during the MCI stage (Zhang et al., 2023). In the AD stage, hippocampal atrophy is a key feature of CI. DKI addresses the limitations of traditional MRI in detecting microscopic structural pathological changes before macroscopic atrophy becomes apparent. Hippocampal MK was the most sensitive single parameter map for differentiating patients with AD, patients with MCI, and cognitively normal individuals (Chu et al., 2022). Another study showed that compared to the NC group, the MCI group exhibited significantly reduced kurtosis values in multiple WM regions (such as the corpus callosum, superior longitudinal fasciculus, and sagittal layer), indicating impaired microstructural complexity, which may be associated with axonal degeneration or myelin damage. Additionally, DKI values in these regions were significantly correlated with MMSE scores, with lower kurtosis values associated with poorer cognitive function, suggesting that DKI parameters could serve as early biomarkers for MCI (Nelson et al., 2024). Research has found that DKI can also be used to explore associations between specific cognitive domain impairments and brain regions, with the corpus callosum commissure associated with visual construction ability, the corpus callosum genu associated with executive function, and the hippocampus associated with memory function (Allen et al., 2019). These findings aid in the stratification of individuals with CI, predict the risk of progression to AD, and provide a powerful tool for studying the neuropathological mechanisms underlying AD (Table 5).

**TABLE 5:** Summary of DTI-derived techniques in PPAD.

DKI				
Authors	Modality	Design	Sample	Main findings
Bergamino et al., 2024	DKI	Cross-sectional	CI = 11 SCD = 10 NC = 12	The mean kurtosis tensor and anisotropy of the kurtosis tensor showed significant differences across the three groups, indicating altered white matter microstructure in CI and SMC individuals. The free water volume fraction (f) also revealed group differences, suggesting changes in extracellular water content.
Zhang et al., 2023	DKI	Cross-sectional	AD = 16 MCI = 37 SCD = 11 NC = 19	In AD vs. NCs, the right hippocampal volume showed the most prominent AUC value (AUC = 0.977); in MCI vs. NCs, the right hippocampal MD was the most sensitive discriminator (AUC = 0.819); in SCD vs. NCs, the left hippocampal MK was the most sensitive biomarker (AUC = 0.775).
Chu et al., 2022	DKI	Cross-sectional	AD = 20 MCI = 21 NC = 20	Precuneus MD, temporal MK, precuneus MK, and hippocampal MK were significantly correlated with neuropsychological test scores. Hippocampal MK showed the strongest correlation with the medial temporal lobe atrophy score (*r* = −0.510), and precuneus MD had the strongest correlation with the Koedam score (*r* = 0.463).
Nelson et al., 2024	DKI	Cross-sectional	MCI = 46 NC = 55	This study found significant differences in white matter integrity, particularly in free water levels and kurtosis values, suggesting neuroinflammatory responses and microstructural integrity disruption in MCI. Moreover, negative correlations between Mini-Mental State Examination (MMSE) scores and free water levels in the brain within the MCI group point to the potential of these measures as early biomarkers for cognitive impairment.
Allen et al., 2019	DKI	Cross-sectional	aMCI = 19	Statistically significant correlations between diffusion metrics and cognitive z-scores were detected: visuospatial-visuoconstructional z-scores only correlated with alterations in the corpus callosum splenium, executive functioning z-scores with the corpus callosum genu, memory testing z-scores with the left hippocampus, and composite z-scores with the anterior centrum semiovale.
**FW-DTI**				
**Authors**	**Modality**	**Design**	**Sample**	**Main findings**
DeSimone et al., 2024	FW-DTI	Cross-sectional	AD = 21 MCI = 80 NC = 27	Plasma + /PET- demonstrated increased FW (24 regions) and decreased FAt (66 regions) compared to plasma-/PET-. FW (16 regions) and FAt (51 regions) were increased in plasma + /PET + compared to plasma + /PET-. Composite brain FW correlated with plasma Aβ42/40 and p-tau181.
Archer et al., 2019	FW-DTI	Cross-sectional	AD = 30 NC = 32	In AD, we found robust between-group differences in FW (31/32 TCATT tracts) in the absence of between-group differences in FAT. FW in the inferior temporal gyrus TCATT tract was most associated with MoCA scores in AD.
Bergamino et al., 2021	FW-DTI	Cross-sectional	AD = 28 NC = 30	Using FW-DTI, improved consistency was observed in FA, AxD, and RD, and the complementary FW index was higher in the AD group as expected. With both standard and FW-DTI, higher values of MA coupled with higher values of FA in AD were found in the anterior thalamic radiation and cortico-spinal tract, most likely arising from a loss of crossing fibers.
Yang Y. et al., 2023	FW-DTI	Cross-sectional	*n* = 1607	Conventional dMRI metrics were associated globally with diagnostic status; following FW correction, the FW metric itself exhibited global associations with diagnostic status, but intracellular metric associations were diminished.
**FW-DTI**				
**Authors**	**Modality**	**Design**	**Sample**	**Main findings**
Sun et al., 2024	FW-DTI	Cross-sectional	AD = 29 MCI = 44 NC = 38	Compared with CH/MCI-n/MCI-p, AD showed significant change in tissue compartment indices of FW-DTI. No difference was found in the FW index among pair-wise group comparisons (the minimum FWE-corrected *P* = 0.114). There was a significant association between FW-DTI indices and memory and visuospatial function.
Pichet Binette et al., 2021	FW-DTI	Cross-sectional	*n* = 303	The study focused on free-water-corrected diffusion measures in the anterior cingulum, posterior cingulum, and uncinate fasciculus in cognitively normal older adults at risk of sporadic AD and presymptomatic mutation carriers of autosomal dominant AD. In Aβ-positive or tau-positive groups, lower tissue fractional anisotropy and higher mean diffusivity related to greater Aβ and tau burden in both cohorts. Associations were found in the posterior cingulum and uncinate fasciculus in preclinical sporadic AD, and in the anterior and posterior cingulum in presymptomatic mutation carriers.
**NODDI**				
**Authors**	**Modality**	**Design**	**Sample**	**Main findings**
Fu et al., 2020	NODDI	Cross-sectional	AD = 14 MCI = 14 NC = 14	Compared with the HC group, the NDI and ODI values decreased significantly and the Viso values were significantly increased in the MCI and AD groups (*p* < 0.01, threshold-free cluster enhancement (TFCE)-corrected); however, there were no significant differences in FA values in the MCI group. The NDI, ODI, and Viso values of multiple fibers were significantly correlated with MMSE and MoCA scores. For the diagnosis of AD, the area under the ROC curve (AUC) for the NDI value of the splenium of corpus callosum was larger than the FA value (AUC = 0.885, 0.714, *p* = 0.042). The AUC of the Viso value of the right cerebral peduncle was larger than FA value (AUC = 0.934, 0.531, *p* = 0.004).
Vogt et al., 2020	NODDI	Cross-sectional	AD = 26 MCI = 30 NC = 56	It demonstrated that neurite density index (NDI) was significantly lower throughout temporal and parietal cortical regions in MCI, while both NDI and orientation dispersion index (ODI) were lower throughout parietal, temporal, and frontal regions in AD dementia. In follow-up ROI analyses comparing microstructure and cortical thickness (derived from T1-weighted MRI) within the same brain regions, differences in NODDI metrics remained, even after controlling for cortical thickness. Moreover, for participants with MCI, gray matter NDI-but not cortical thickness-was lower in temporal, parietal, and posterior cingulate regions.
Wen et al., 2019	NODDI	Cross-sectional	MCI = 22 SCD = 38 NC = 40	Lower DTI fractional anisotropy and higher radial diffusivity were observed in the cingulum, thalamic radiation, and forceps major of participants with MCI. These tracts of interest also had the highest predictive power to discriminate groups. Diffusion metrics were associated with cognitive performance, particularly Rey Auditory Verbal Learning Test immediate recall, with the highest association observed in participants with MCI.

#### 3.2.2 Application of FW-DTI

Diffusion tensor imaging (DTI) assumes that each voxel contains a single tissue compartment; however, the presence of extracellular free water introduces a partial volume effect, significantly affecting the accuracy of DTI measurements (Kamagata et al., 2021). FW-DTI is a dual-tensor model (intracellular diffusion + free water diffusion) that eliminates the influence of free water contamination compared to standard DTI techniques. FW-DTI can calculate the extracellular free water fraction (FWF) to exclude the influence of free water. Free-water-corrected DTI metrics primarily include free-water-corrected FA (FAt), free-water-corrected MD (MDt), free-water-corrected AD (ADt), and free-water-corrected RD (RDt).

Researchers recruited NC participants who were Aβ-PET negative but Aβ42/40 positive and found that plasma Aβ42/40 positivity was associated with increased extracellular FWF and reduced tissue FAt in multiple brain regions (DeSimone et al., 2024). FW-DTI detected changes in WM microstructure before the brain reached the Aβ-PET positivity threshold, indicating its high sensitivity for early AD monitoring. Multiple studies found elevated FEW in widely distributed WM tracts in MCI and AD brains compared to NC (Archer et al., 2019; Bergamino et al., 2021). A large-scale study found that increased clinical severity along the AD continuum was associated with elevated FWF in the marginal tracts, particularly within the calcarine and cingulate gyrus (Yang Y. et al., 2023), and that elevated FWF was associated with poorer attention, executive function, cognitive performance, and visuospatial structure (Sun et al., 2024), indicating an association between FWF and clinical diagnostic severity. FW-DTI scans of NC elderly individuals carrying mutation genes prior to symptom onset showed that lower FAt, higher MDt, and RDt in the anterior cingulate gyrus and uncinate fasciculus were associated with higher pathological burden, while standard DTI metrics, except for FA, were not significant (Pichet Binette et al., 2021). Compared to DTI, FW-DTI can more early reveal the association between WM microstructural changes and AD pathology. In summary, FW-DTI demonstrates better tissue specificity in characterizing brain WM and higher sensitivity in detecting brain microstructural changes. Additionally, FW-DTI has the potential to reflect AD pathological changes (Table 5).

#### 3.2.3 Application of NODDI

Neurite direction dispersion and density imaging (NODDI) is a multi-compartment biophysical model capable of distinguishing the different diffusion patterns of water molecules in three compartments: within neurites, outside neurites, and CSF (Zhang et al., 2012). It is used to assess the microstructural changes in tissue (Winston et al., 2020). The parameters provided by NODDI include the neurite density index (NDI), the dispersion index (ODI), and the volume fraction of isotropic water diffusion (Viso) in the CSF. NDI represents the volume fraction of neurites, while ODI represents the curvature and branching degree of neurites, enabling separate analysis of neurite density and dispersion, thereby providing a more precise description of complex tissue structural changes (Chung et al., 2016).

Studies based on NODDI have found that, compared to the NC group, the NDI and ODI values were significantly reduced in the MCI and AD groups, while the Viso values were significantly increased. However, there were no significant differences in FA values, indicating that NDI is more sensitive to changes in WM microstructure than FA (Fu et al., 2020). In addition to detecting WM microstructural changes, NODDI can also be used to observe gray matter abnormalities. In MCI, NDI values were significantly reduced in the entire temporal and parietal cortex regions, while in AD dementia, NDI and ODI values were reduced in the entire parietal, temporal, and frontal regions (Vogt et al., 2020). Furthermore, NODDI also has classification and predictive value. By combining DTI and NODDI metrics, a study using LASSO regression analysis found that WM changes in the parahippocampal gyrus, posterior thalamic radiation, and corpus callosum commissure region have high predictive ability for distinguishing MCI from CN or SCD (Wen et al., 2019). A meta-analysis revealed that microstructural changes in MCI/AD are characterized by reduced fiber orientation dispersion, decreased neurite density, and increased fiber orientation dispersion in specific WM tracts (including the cingulum, uncinate fasciculus, and left posterior thalamic radiation) within the hippocampus. Additionally, reduced NDI is associated with cognitive decline in MCI/AD patients (Zhong et al., 2023; Table 5).

Diffusion MRI is widely used to detect microstructural changes in brain tissue associated with cerebral pathologies and ageing. Nevertheless, dMRI is still limited by some technical difficulties, such as b value selection and a long acquisition time. Diffusion MRI is very sensitive to motion, as phase shifts induced microscopically by movement of the head, cardiac pulsation and breathing can affect the results. This sensitivity increases with the intensity and duration of gradient pulses, which are defined by the b values. During image acquisition, a strong gradient leads to small brain tissue movements, and phase offsets will occur, inducing signal dropouts in the diffusion images (Soares et al., 2013). However, these disadvantages do not appear to have affected the development and application of dMRI techniques. An emerging technology combining diffusion imaging and peptide labeling, which showed tolerance to nearly 30° of rotational motion, provides highly robust diffusion and relaxometry data and offers potential for future applications in diffusion-relaxometry multi-compartment modeling (Fair et al., 2021). With further developments in MRI technology and optimization of postprocessing technology, dMRI is expected to undergo a wide range of advancements.

## 4 Multimodal MRI applications

Single-modality imaging methods are limited in their ability to evaluate complex and progressive diseases. Structural MRI can only provide structural information such as volume and cortical thickness, while DTI can only provide diffusion information of water molecules. Dementia is a heterogeneous disease, which limits the explanatory power of a single biomarker. Multimodal magnetic resonance imaging, by combining sMRI, functional MRI, diffusion imaging and metabolic imaging, can comprehensively reveal the neurodegenerative process of AD. For example, hippocampal atrophy may be accompanied by abnormal functional connectivity of the default network mode (Grieder et al., 2018), while white matter fiber damage may occur earlier than cortical atrophy (Feng et al., 2021). This multi-dimensional information complementarity can not only improve the sensitivity and specificity of early diagnosis, but also distinguish AD subtypes (such as amnestic type and non-amnestic type), providing a basis for individualized treatment. Thus, multimodal magnetic resonance has been widely investigated and applied in the past 10 years. Below, we summarize the development of multimodal MRI and its clinical application in SCD and MCI.

### 4.1 Multimodal MRI in SCD

A growing number of studies have combined multimodal neuroimaging techniques with PET and MRI to observe AD-induced pathological changes in SCD patients (Chételat, 2018). Abnormal amyloid deposition has been identified as a key factor in triggering downstream neurodegenerative cascade responses (Chen et al., 2024; Song et al., 2022; Yan et al., 2024). Between-group analyses revealed that SCD subjects with higher levels of amyloid deposition presented with significantly greater gray matter atrophy than those with lower levels of amyloid deposition (Chételat et al., 2010). A disease severity index has been developed from multivariate analyses involving both amyloid PET and structural MRI; this index may identify individuals with AD-like patterns of SCD as an appropriate risk population (Ferreira et al., 2017). In addition, researchers have combined amyloid PET, FDG-PET, and structural MRI data to identify pathological patterns corresponding to different points along the AD continuum. The results revealed three different imaging biomarker patterns present in patients with different stages of AD (Wirth et al., 2018). A longitudinal study indicated that reductions in cognitive performance over time were associated with cerebral hypometabolism in the precuneus at baseline without gray matter atrophy according to the joint use of FDG-PET and MRI (Scheef et al., 2012). Patients with clinical SCD have been shown to have greater progression of gray matter atrophy over time than patients with community SCD, suggesting that clinical SCD may represent a greater risk for dementia due to AD (Kuhn et al., 2019).

In addition to observing cerebral WM degeneration in SCD patients, DTI can be used to study structural and functional changes in the brain of SCD patients in depth from multiple perspectives when combined with other techniques. The accumulation of β-amyloid Aβ and tau neurofibrillary tangles in the brain is a typical pathological feature of AD (Scheltens et al., 2021). Thus, studies of MCI and SCD patients with DTI combined with tau PET have confirmed a link between tau protein abnormalities and WM degeneration (Wen et al., 2021). Interactions between gray and WM neurodegeneration can also be identified with sMRI combined with DTI, confirming that more subjective cognitive complaints are associated with smaller volumes of the hippocampus, frontal lobes, temporal lobes and insula and an increased WM MD (Cedres et al., 2021). Combined fMRI and DTI analyses revealed that brain connectivity and excitability and WM integrity were significantly lower in patients with SCD than in NCs (Fogel et al., 2021). In another study, the accuracy of DTI in discriminating β-amyloid-positive patients with MCI from β-amyloid-negative controls was 80%. However, patients with SCD only presented with spatially restricted WM alterations in the anisotropic modality, and DTI lacks the ability to identify β-amyloid-positive and β-amyloid-negative patients with SCD (Teipel et al., 2019). The studies of multimodal MRI in SCD also been summarized in Table 6.

**TABLE 6:** Summary of multimodal magnetic resonance in SCD.

Authors	Modality	Design	Sample	Main findings
Chételat et al., 2010	MRI Aβ-PET	Cross-sectional	SCD+ = 19 SCD− = 30 MCI+ = 22 AD+ = 34 NC+ = 13 NC− = 31	In SCD subjects, individuals with higher levels of amyloid deposition showed significant gray matter atrophy compared to those with lower levels of amyloid deposition.
Ferreira et al., 2017	MRI Aβ-PET	longitudinal study	SCD = 86 MCI = 45 AD = 38 NC = 69	The disease severity index identified eleven (13%) SMD individuals with an AD-like pattern of brain atrophy. These individuals showed lower cognitive performance, increased CDR-SOB, higher amyloid burden and worse clinical progression (6.2 times higher likelihood to develop MCI, dementia or die than healthy controls).
Wirth et al., 2018	MRI Aβ-PET FDG-PET	Cross-sectional	APOE4 = 17 SCD = 16 MCI = 30	In mild cognitive impairment patients, 3 distinct biomarker patterns were recovered, similarly seen in AD patients: (1) in medial temporal regions, local GMV reduction exceeded hypometabolism, (2) in temporoparietal regions, hypometabolism predominated over GMV reduction, and (3) in frontal regions, Aβ deposition exceeded GMV reduction and hypometabolism. In subjective cognitive decline patients, only pattern 1 was detected, while APOE4 carriers demonstrated only pattern 3.
Kuhn et al., 2019	MRI Aβ-PET FDG-PET	longitudinal study	SCD-community = 23 SCD-clinic = 27 NC = 28	Compared to SCD-community, SCD-clinic showed higher informant-reported SCD, depression score, and atrophy progression over time but similar brain amyloid load.
Wen et al., 2021	MRI DTI Tau-PET	Cross-sectional	SCD = 22 MCI = 18 NC = 22	Tau-related WM degeneration is characterized by an increase in the mean diffusivity (with a dominant change in the radial direction) and a decrease in the intra-axonal volume fraction.
Cedres et al., 2021	MRI DTI	Cross-sectional	SCD = 123 NC = 102	A higher number of complaints was associated with reduced hippocampal volume, cortical thinning in several frontal and temporal areas and the insula, and higher MD across the WM skeleton, with a tendency to spare the occipital lobe. SCD-related cortical thinning and increased MD were associated with each other and jointly contributed to complaints, but the contribution of cortical thinning to the number of complaints was stronger.
Fogel et al., 2021	MRI DTI fMRI	Cross-sectional	SCD = 17 aMCI = 12 AD = 11 NC = 15	Both DELPHI analysis of network function and DTI analysis detected a significant decrease in connectivity, excitability, and WM integrity in the SCD group compared to healthy control (NC) subjects; a significant decrease was also noted for aMCI and Dementia groups compared to NC. In contrast, no significant decrease was observed in GM volume in the SCD group compared to healthy norms, a significant GM volume decrease was observed only in objectively cognitively impaired aMCI subjects and in dementia subjects.
Teipel et al., 2019	DTI Aβ-PET	Cross-sectional		DTI was 80% accurate in discriminating Aβ-positive cases of MCI from Aβ-negative controls, in contrast to patients with SCD who showed spatially-restricted white matter alterations in the anisotropic modality only, and DTI’s discrimination between Aβ-positive and negative cases of SCD was No value

### 4.2 Multimodal MRI in MCI

A study combining 18F-flortaucipir PET and sMRI revealed that the whole-brain 18F-flortaucipir standardized uptake ratio (SUVr) was significantly correlated with the levels of cerebrospinal fluid t-tau and p-tau; the strongest correlations were found in temporal regions (as confirmed with voxel analyses), suggesting that PET is a useful tool for visualizing pathological changes in AD. The metrics derived from 18F-flortaucipir PET were also associated with CSF β-amyloid levels, situational memory, visuospatial working memory, and cortical thickness, regardless of hippocampal volume (Okafor et al., 2020). Another study, involving the use of 18F-FDG PET/MRI (specifically T1-weighted MRI), revealed that the posterior cingulate cortex and precuneus demonstrated reduced metabolic activity in patients with MCI and AD, whereas the atrophy in these regions was not significant. These results suggest that the uptake of 18F-FDG in these two regions may be a marker for early AD (Bailly et al., 2015). A study combining automated MRI-based brain volume measurements and THK-5351 PET revealed that the isthmus of cingulate gyrus and IPL are important regions for distinguishing AD patients from NCs and MCI patients, suggesting the potential discriminatory value of this combined method (Kim et al., 2021). Other researchers have combined MRI and PET/CT for head-to-head multimodal imaging with 18F-FDG and 18F-AV45 to visualize and quantify brain morphology, glucose metabolism, and amyloid levels, identifying the AV45/FDG/NVol index as a novel quantitative molecular imaging biomarker associated with the clinical neurocognitive status of the patient (Thientunyakit et al., 2020).

Another study based on sMRI and fMRI revealed a significant reduction in gray matter volume in frontotemporal parietal structures as well as impaired resting functional connectivity in the default mode network (DMN) and executive network in patients with aMCI. Moreover, the abovementioned methods were reported to distinguish aMCI patients from healthy ageing individuals, and the results were considered to constitute a neuroimaging marker of early dementia (Bharath et al., 2017). The use of 18F-PI-2620 PET and T1-weighted MRI for MCI patients with positive β-amyloid-PET visual findings and follow-up for 1 year revealed that increased tau deposition in the fusiform gyrus was associated with high levels of p-tau and t-tau in the CSF. However, a low hippocampal volume was associated with increased tau loading at baseline, with a significant increase in tau loading observed in the temporal cortex, fusiform gyrus, and inferotemporal cortex after 1 year; however, the change in amyloid load was not significant (Bullich et al., 2022).

Diffusion tensor imaging (DTI) and resting-state fMRI were combined to assess brain function in AD and MCI patients and NCs. Patterns of interhemispheric functional connectivity were measured with voxel-mirror-homotopic connectivity (VMHC), and a reduction in VMHC was observed in forebrain regions, including the prefrontal cortex and subcortical regions, of AD and MCI subjects. DTI analysis further revealed that the most significant difference among the three cohorts was in the FA in the genu of the corpus callosum, which was positively correlated with VMHC in the prefrontal and subcortical regions. In addition, the diffusion parameters in the aforementioned brain regions were significantly correlated with metrics of cognitive performance. These results suggest a specific pattern of interhemispheric functional connectivity changes in AD and MCI patients that is significantly associated with changes in the structural integrity of the WM along the midline (Wang Z. et al., 2015). We have summarized the studies of multimodal MRI in MCI in Table 7.

**TABLE 7:** Summary of Multimodal magnetic resonance in MCI.

Authors	Modality	Design	Sample	Main findings
Okafor et al., 2020	MRI Tau-PET	Cross-sectional	*N* = 20	18F-flortaucipir PET was also associated with CSF Aβ, episodic memory, visuospatial working memory, and brain cortical thickness but not hippocampal volume.
Bailly et al., 2015	MRI FDG-PET	Cross-sectional	MCI = 17 AD = 17 NC = 13	It found graduated hypometabolism in the posterior cingulate cortex and the precuneus in prodromal AD (MCI) and AD, whereas atrophy was not significant.
(Kim et al., 2021	MRI Tau-PET	Cross-sectional	MCI = 55 AD = 26 NC = 32	The cingulate isthmus and inferior parietal lobule as significant regions in discriminating AD from NC and MCI.
Thientunyakit et al., 2020	MRI Aβ-PET FDG-PET	Cross-sectional	MCI = 31 AD = 33 NC = 20	A significant direct linear correlation was observed between the AV45/FDG/NVol index and ADAS-Cog test score and an inverse correlation with TMSE score at baseline and with the degree of changes in ADAS and TMSE scores assessed 1 year later (disease progression).
Bharath et al., 2017	MRI DTI fMRI	Cross-sectional	MCI = 48 NC = 48	It concludes that the demonstrable neuroimaging findings in aMCI include significant gray matter volumetric reductions in the fronto-temporo-parietal structures as well as resting state functional connectivity disturbances in DMN and executive network.
Bullich et al., 2022	MRI Aβ-PET Tau-PET	longitudinal study	*N* = 74	Increased tau deposition in the fusiform gyrus was associated with high CSF p-tau and t-tau, and low hippocampal volume was associated with increased tau loading at baseline, with a significant increase in tau loading in temporal cortex, fusiform gyrus, and inferotemporal cortex after 1 year, yet there was no significant difference in amyloid load change.
Wang Z. et al., 2015	fMRI DTI	Cross-sectional	MCI = 16 AD = 16 NC = 16	Decreased VMHC was observed in AD and MCI subjects in anterior brain regions including the prefrontal cortices and subcortical regions with a pattern of AD < MCI < CN. Increased VMHC was observed in MCI subjects in posterior brain regions with patterns of AD/CN < MCI (sensorimotor cortex) and AD < CN/MCI (occipital gyrus). DTI analysis showed the most significant difference among the three cohorts was the fractional anisotropy in the genu of corpus callosum, which was positively associated with the VMHC of prefrontal and subcortical regions. Across all the three cohorts, the diffusion parameters in the genu of corpus callosum and VMHC in the above brain regions had significant correlation with the cognitive performance.

Risk genes also have an impact on the structure of the brain. A study examined cross-sectional AD biomarkers for participant with MCI (*n* = 930), which found number of ApoEε4 alleles was associated with smaller hippocampal and amygdala volumes and poorer cognition (Hobel et al., 2019). BIN1, a key AD susceptibility gene after APOE, has higher expression in AD. After adjusting for confounding factors, the association between the BIN1 rs10200967 genotype and left hippocampal-amygdaloid transition area atrophy significant in the MCI (Luo et al., 2025). The follow-up study found that CTCF, UQCR11 and WDR5B gene had the most significant correlations with left paracentral lobule and sulcus and right subparietal sulcus thickness (Wang et al., 2021). In addition to exploring morphological, functional and pathological changes in the brain, MRI can compare the impact of genetic factors on the brain among different populations.

With the emergence of β-amyloid-PET and tau-PET, imaging observations of AD have shifted to the molecular pathological level. Furthermore, the combination of PET and MRI has provided a new way to spatially visualize pathological changes in AD and more data on the pathogenesis associated with PPAD. Thus, multimodal neuroimaging techniques have shown great advantages in exploring the relationships among different AD biomarkers, but further multimodal neuroimaging studies of SCD are needed.

## 5 Summary

**FIGURE 1:**
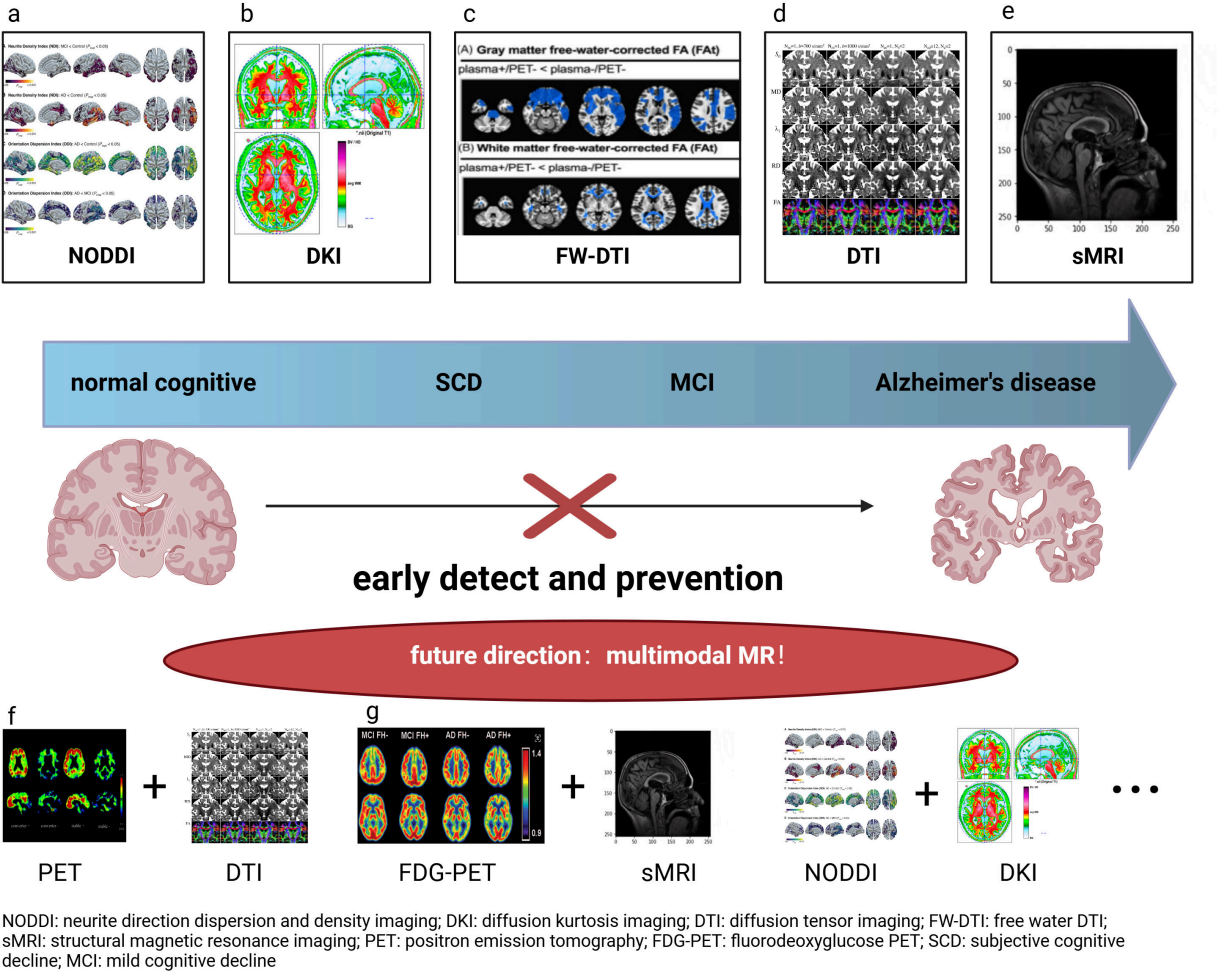
The application of multimodal MRI in evaluation of cognitive Function. Panel **(a)** is based on Vogt et al. (2020). Panel **(b)** is based on Zhang et al. (2023). Panel **(c)** is based on DeSimone et al. (2024). Panel **(d)** is based on Teipel et al. (2014). Panel **(e)** is based on Balasundaram et al. (2023). Panel **(f)** is based on Hatashita and Yamasaki (2013). Panel **(g)** is based on Mosconi and McHugh (2011). NODDI, neurite direction dispersion and density imaging; DKI, diffusion kurtosis imaging; DTI, diffusion tensor imaging; FW-DTI, free water DTI; sMRI, structural magnetic resonance imaging; PET, positron emission tomography; FDG-PET, fluorodeoxyglucose PET; SCD, subjective cognitive decline; MCI, mild cognitive decline.

However, there are still differences between SCD and MCI in neuroimaging. In terms of macroscopic changes, the differences mainly lie in the degree of hippocampal atrophy and the extent of cortical thinning. Nevertheless, current studies have not reached a consensus on the sMRI parameters for distinguishing between SCD and MCI. Large-scale real-world studies are needed to establish specific thresholds. In terms of white matter fiber tract damage, both SCD and MCI exhibit defects in the hippocampus and parahippocampal gyrus, but they also show differences in several other brain regions. For example, SCD involves changes in the LOC and insula, while MCI presents defects in the calcarine and cingulate gyrus (Figure 1). These differences may involve neural damage and compensatory mechanisms, which require further validation through additional basic and clinical studies.

Structural and diffusion tensor imaging techniques have shown great potential in investigating, both qualitatively and quantitatively, the pathogenesis, early diagnosis, disease progression, and treatment efficacy in the PPAD. However, current research in this field has numerous limitations associated with technological developments and rapid changes in imaging methods. First, novel diffusion, functional and perfusion techniques as well as artificial intelligence techniques have not yet been widely used in preclinical studies or clinical practice involving AD. Second, existing MRI studies are mostly limited by small-sample, single-center, cross-sectional designs and a primary focus on single-modality MRI analyses, thus limiting the generalizability and reliability of the results. Third, the existing single-modality MRI analyses have low sensitivity and specificity and lack both a pathological basis and a uniform threshold for quantitative measurements. Therefore, future studies should focus on integrating advanced technologies, increasing the sample size, adopting a multicentre, longitudinal tracking design, and integrating multimodal imaging and artificial intelligence for more comprehensive analyses to reveal the pathological mechanisms underlying preclinical neurological changes in AD. Continued development and modification of magnetic resonance technology are important and will aid in the wider and more reliable application of multimodal MRI to each stage of AD in research and clinical practice.
